# Guidance, Navigation and Control for Autonomous Quadrotor Flight in an Agricultural Field: The Case of Vineyards

**DOI:** 10.3390/s22228865

**Published:** 2022-11-16

**Authors:** Adel Mokrane, Abdelaziz Benallegue, Amal Choukchou-Braham, Abdelhafid El Hadri, Brahim Cherki

**Affiliations:** 1 Laboratoire d’Ingénierie des Systèmes de Versailles, Université Paris-Saclay, UVSQ, LISV, 78124 Vélizy-Villacoublay, France; 2 Laboratoire d’Automatique de Tlemcen, University of Tlemcen, Tlemcen 13000, Algeria

**Keywords:** guidance, navigation, trajectory generation, quadrotor, re-planning, trajectory tracking controller

## Abstract

In this paper, we present a complete and efficient solution of guidance, navigation and control for a quadrotor platform to accomplish 3D coverage flight missions in mapped vineyard terrains. Firstly, an occupancy grid map of the terrain is used to generate a safe guiding coverage path using an Iterative Structured Orientation planning algorithm. Secondly, way-points are extracted from the generated path and added to them trajectory’s velocities and accelerations constraints. The constrained way-points are fed into a Linear Quadratic Regulator algorithm so as to generate global minimum snap optimal trajectory while satisfying both the pointing and the corridor constraints. Then, when facing unexpected obstacles, the quadrotor tends to re-plan its path in real-time locally using an Improved Artificial Potential Field algorithm. Finally, a geometric trajectory tracking controller is developed on the Special Euclidean group SE(3). The aim of this controller is to track the generated trajectory while pointing towards predetermined direction using the vector measurements provided by the inertial unit. The performance of the proposed method is demonstrated through several simulation results. In particular, safe guiding paths are achieved. Obstacle-free optimal trajectories that satisfy the way-point position, the pointing direction, and the corridor constraints, are successfully generated with optimized platform snap. Besides, the implemented geometric controller can achieve higher trajectory tracking accuracy with an absolute value of the maximum error in the order of $10^{-3}$ m.

## 1. Introduction

A century ago, technology was introduced to agriculture; the first tractor was launched in 1913 [1]. Nowadays, mechanical automated machines have been extensively used in many agricultural tasks. This has led to increased productivity, and has reduced the amount of human labor. This may not be sufficient to satisfy the world’s need for food, yet. Hence, in the beginning of the 1990s, the concept of Precision Agriculture (PA) was created. PA consists of specific management practices based on observation, measurement and response to inter and intra-field crop variability [2]. In PA, farmers and growers are able to increase productivity with quality by the means of agricultural systems in which the site-specific management practice, that divides the agricultural fields into zones or blocks, is implemented at the right place, with the right intensity, and at the right time [3]. This is particularly true in viticulture where it is possible to differentiate between zones of different quality of grapes and perform proper management. Precision Viticulture (PV) is a cyclic management approach that aims to use the available developed technologies for maximizing the oenological potential and optimizing agronomic inputs in the vineyard to minimize the costs and protect environmental sustainability.

Many regional strategic research agendas have been developed in the recent years. Most of them state that the use of automated robotic platforms will result in food security and improve agriculture efficiency. Although many research approaches have been conducted, few commercial solutions have come into existence recently [4]. Pruning, spraying, planting, harvesting, monitoring, and remote sensing are known as the field operations of such approaches where autonomous navigation is critical. Autonomous navigation, in general, comprises localization, mapping, motion control and path and trajectory planning. This latter is the crucial part to insure safe and efficient robot navigation in any environment.

Path and trajectory planning is an essential tool for automation and optimization of the aforementioned field operations. It is used to achieve a complete guidance, navigation and control (GNC) of automated machines during the execution of the field operation [5]. The field of path planning can be categorized mainly in two major aspects: the point-to-point optimization path planning and the coverage optimization path planning. In recent years, researchers and experts have concentrated their research on the first aspect; however, the study of the second one has relatively reduced.

Most of coverage optimization path planning algorithms deal with 2D field terrains. The aim of these algorithms is to optimally generate driving angles and sequence of tracks of 2D paths so that the field operations can be executed while reducing maneuvering over the field terrain and total time of the operation. Consequently, this may result in reducing soil compaction and fuel consumption. 2D coverage path planning approaches take into consideration that most of the agricultural fields are flat. However, ignoring the elevation changes affects the optimization process, which leads to an inadequate coverage path design [6]. The agricultural field characteristics have a substantial effect on the design and optimization of the coverage path approaches.

Vineyards terrain is an example of an intricate, unstructured, and fickle agricultural fields which can be challenging for robotic machines. Due to their steep slopes and limited room for maneuvering, the need to develop specific path and trajectory planning systems arises. Robotics for vineyards have been well investigated in the literature. Several applications, such as trunk recognition and localization [7], pruning [8], yield estimation [9] and irrigation [10], have been developed. Different algorithm approaches were used to perform the applications tasks. Graph-based approaches using A* and Dijkstra to solve navigation within the vineyard rows have been presented in [11,12,13]. Most of the algorithms were designed for Unmanned Ground Vehicles (UGVs). These latter have a higher autonomy for covering long distances and offer the benefit of carrying heavier sensor payloads for taking a variety of measurements simultaneously. However, due to their size, weight and intense usage, UGVs have drawbacks of soil compaction causing fertility problems. Besides, vineyards, which are planted in line rows with steep slope hills and/or plowed soil, represent higher complex conditions for UGVs. This has led researchers to develop efficient navigation solutions within vineyard lanes using UAV systems. To the best of our knowledge, few research studies have been proposed in this regard. The researchers in [14] reported a feasible approach to survey vineyards using UAVs. The approach described how navigation missions were achieved in three steps: offline definition of the workspace, computation of coverage paths, and mission execution. The experimental implementation of the approach was conducted and a brief analysis of the results was highlighted in [15]. In [16,17], a UAV was exploited perform in-field operations. An inter-row vine navigation strategy was built by combining a Rapidly-exploring Random Tree (RRT*) global path planner, a Dynamic-Window Approach (DWA) local path planner and a Model Predictive Control (MPC) trajectory tracker. Table 1, which summarizes the proposed approaches, reports that none of these approaches offer complete solutions to solve the autonomous navigation problem for UAVs in a vineyard.

The problem of 3D autonomous GNC for aerial vehicles in the agricultural field is very challenging and complex. In general, the problem cannot be solved directly. It can be decomposed into a multi-phase sub-problems: path planning, trajectory optimization, trajectory re-planning and trajectory tracking. Owing to the numerous challenges of each sub-problem, a feasibility screening among the possible already known technologies is conducted. In this paper, we attempt to design a complete and efficient solution, which will be tested in a robotic simulator, based on a combination of algorithms used for the first time in a PA scenario. These algorithms are accurately selected among those currently available in the literature aiming to identify the best combination of GNC algorithms. The solution is developed for 3D autonomous flying in vineyards. The proposed method generates optimal trajectories that allow the quadrotor to navigate within the vines rows while pointing toward them, simultaneously, for sake of performing remote sensing. An offline definition of the optimal trajectory is used. This task is typically performed in two consecutive steps. The first makes use of artificial Occupancy Grid Maps (OGMs) of the vineyards for generating safe coverage paths between the vines rows. The purpose of this step is to locate reference way-points with the aid of Digital Elevation Models (DEMs) of the vineyards terrain. The second step uses the extracted way-points to generate global paths that satisfy position, velocity and acceleration constraints at these way-points. Taking inspirations from [18], a Linear Quadratic Regulator (LQR) approach that minimizes the snap and treats the constraints at the way-points as soft, is adopted. In this way, the approach guarantees both a relaxation on satisfying the way-points constraints and a generation of stable position trajectories. When unknown obstacles are detected, an Artificial Potential Field (APF) re-planning strategy commands the UAV to move away from the obstacles. The UAV takes the current position (when obstacle detection occurs) as the start configuration and the next turning point in the global path as the target configuration. Afterwards, a geometric tracking controller, which is inspired from [19], is constructed to follow the predefined trajectories while simultaneously pointing towards prescribed direction (the vines in our case) along the whole flight. The control scheme is developed such that the vehicle orientation is reconstructed from the body measurement vectors provided by the Inertial Measurement Units (IMUs). Although the algorithms used were collected from the literature, minimum contributions were provided in our manuscript. These contributions were reported mainly in the trajectory optimization and the trajectory tracking phases:

1. An offline LQR position trajectory generator that satisfies not only the position and the acceleration constraints but even the velocity ones is designed. In this way, the corridor constraint, which keeps the quadrotor flying within the vine rows, is taken into consideration.
2. A new attitude control law that helps the quadrotor generate its attitude trajectory online using raw vector measurements is implemented. These vectors enable us to avoid the use of the desired attitude directly in the control law, as presented in most existing methods. The control law is used in a geometric trajectory controller that tracks the generated position and attitude trajectories.

The remainder of this paper is organized as follows. In Section 2, we present in detail the proposed GNC method used for the quadrotor. In Section 3, results are emphatically provided. Section 4 discusses the findings of Section 3. Finally, the conclusion and further work are drawn in Section 5.

## 2. Materials and Methods

**Figure 1:**
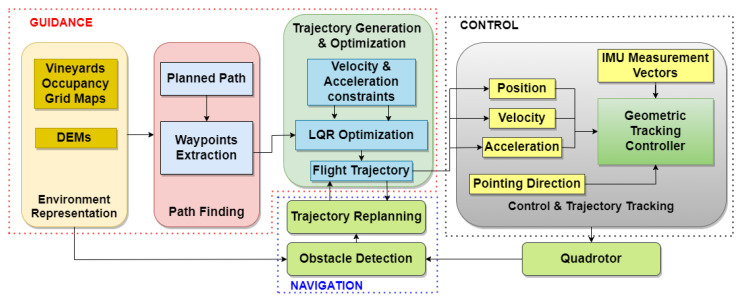
An overview of the GNC global structure.

Broadly speaking, the UAV flight trajectory generation is divided into two modules: the initial path finding and the trajectory generation and optimization. The goal of the initial path finding is to plan passable collision-free paths. Due to the system dynamic feasibility and safety constraints, it is undesirable to provide the generated paths directly to the UAV in order to be executed. The trajectory generation and optimization aims to optimize the path from the former module while satisfying the vehicle dynamics feasibility. In addition, this module helps to achieve flight trajectories that guarantee higher degree of safety. In order to achieve safe and efficient offline flights, OGM and DEM representations of the vineyard environments are used. Figure 1 depicts on overview of the workflow used in this paper.

The workflow above is mainly divided into five parts: UAV terrain mapping, path finding, trajectory generation and optimization, trajectory re-planning, and control and trajectory tracking.

### 2.1. UAV Terrain Mapping

Mapping is the process of modelling the robot’s environment. It uses the observations from the robot’s onboard sensors for constructing a consistent environment model called maps [9]. Maps are used by robotics vehicles for locating themselves and making motion plans. Aerial terrain mapping, which can be achieved using an UAV, is an essential feature for autonomous vehicles to perform navigation in unknown environments. The UAV acquires information about the terrain geometry and the existence of static and dynamic obstacles. In the literature, there are mainly three types of maps: grid maps [20], feature maps [21], and topological maps [22].

Occupancy Grid Maps (OGMs) are the mostly utilized type for robot’s environment representation. They are simple and easy to maintain. They are used to store the obstacle information for sake of planning safe and stable geometric paths. OGMs decompose the environment into a grid of cells, each one either occupied, so that the agent cannot pass through it, or free, so that the agent can traverse it. OGM cannot be accurate; nevertheless, by selecting a small enough cell size they can offer all the necessary information. An example of OGM is the binary OGM that is adopted in this work where each cell is a binary random variable; 1 for free space and 0 for obstacle space (the vines in our case).

Another type of grid maps are Digital Elevation Maps (DEMs). They exhibit the relief of the terrain in digital format at regularly spaced horizontal intervals. DEMs are used in several applications like geospatial applications, 3D graphics displaying, terrain aspect, slope, and terrain profiles between selected points. They are sometimes referred to as Digital Height Model (DHM) where the square grids are organized in columns and rows where each grid point refers to the height at that location.

### 2.2. Coverage Path Finding

Coverage path finding is the first step of trajectory generation. Coverage path finding consists of finding the path that covers any environment, while taking the motion restrictions and avoiding the collision with the obstacles present in that environment [23]. This step is computationally complex; hence, approximate or even heuristic solutions are used for solving it. The environment is dividing into sub-regions, selecting a sequence of those sub-regions and then generating a path that traverses each sub-region in turn. Such methods take for granted the convex polygonal environment and perform exact cell decomposition, which is less time consuming, and the robot dimensions have exactly the same dimensions of one cell within the grid map. Several conditions are considered when performing coverage path finding [24]:

1. The robot must cover completely all points in its environment.
2. The robot must fill the environment without overlapping routes.
3. Continuous and sequential coverage process without repetition of the routes is entailed.
4. The robot must avoid obstacles (if present).
5. Motion routes, which are simple (straight lines or circles), must be used (for sake of simplicity).
6. Optimal path is obtained under predefined conditions.

However, taking into considerations all the above conditions in a complex environment is unfeasible in most situations.

This paper adopts the Iterative Structured Orientation Algorithm (ISOA) proposed in [25]. The ISOA is an optimal approach where its optimality is delineated in terms of the distance of the generated path. The reason behind choosing such an algorithm is that it makes use of OGMs and plans a complete coverage by using the approach of main lines. These are a beam of parallel lines with an orientation in the grid map. Straight lines with maximum length, which are bordered by the map and interrupted by the obstacles, are guaranteed by that orientation. The optimal continuous coverage path is achieved by connecting the beam of lines.

**Figure 2:**
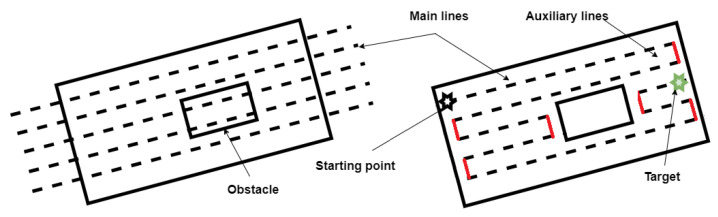
The map with both main lines (black) and auxiliary lines (red).

Given a modeled map $M={\left[M_{ij}\right]}_{i=1..n,j=1..m}\in R^{n\times m}$ as shown in Figure 2, where *n* is number of horizontal pixels and *m* is the number of vertical pixels with

(1) $$M_{ij}=\left\{\begin{array}{c} 1,if\;pixel(i,j)white\\ 0,if\;pixel(i,j)black \end{array}\right.$$

The problem of finding an optimal path can be summarized as follows

1. Find the appropriate beam of parallel lines and their orientation;
2. Get the main segments;
3. Connect these segments with the auxiliary segments to form a continuous optimal coverage path.

**Figure 3:**
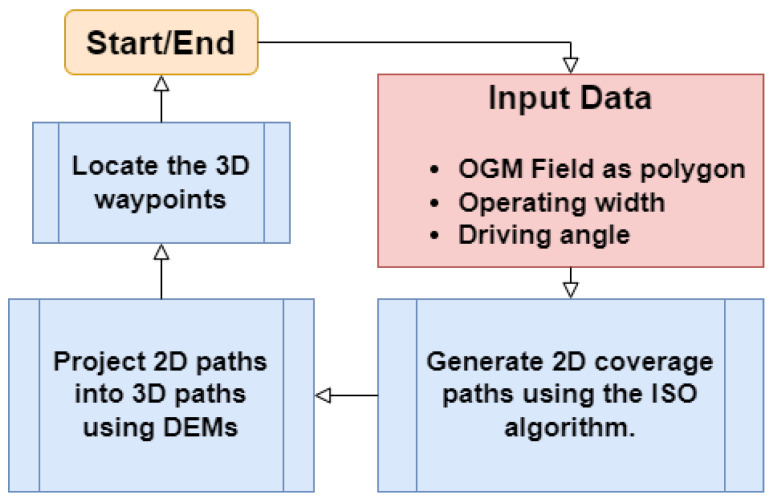
The 3D local goals extraction flowchart.

The optimal path is used then for extracting the 2D local goals (way-points). Finally, by the use of the DEMs, the position along the z-axis is added to the way-points. The result of this step is a set of 3D local goals *W* required for trajectory generation. The flowchart of the approach is shown in Figure 3.

### 2.3. Trajectory Generation and Optimization

Trajectory generation algorithms are core problems in UAV control which allow them to attain their full autonomy. They can be categorized mainly into three classes. The algorithms in the first class can be regarded as primarily geometric. The process of planning a trajectory comprises a geometric path generation, first. Thereafter, the path is time parameterized while satisfying the UAV dynamic constraints. In this case, path primitives like lines [26] polynomials [27] and splines [28] are considered. In the second class, the algorithms are used for planning trajectories while minimizing a certain derivative of the UAV position (or combinations thereof). The differential flatness property of the UAV is used in such a way the trajectory feasibility depends on the derivatives. Here, minimum snap trajectory generation [29], weighted sum of derivative minimization [30], maneuver duration minimization [31] can be listed. Model predictive algorithms fall under this class. For instance in [32], a Learning Based Model Predictive Control (LBMPC) is implemented with a priori unknown trajectory. The algorithm showed its robustness, safety and convergence while used for catching a thrown ball. In the third class, the algorithms are used to solve an optimal control problem to generate numerical trajectories based on the full vehicle system dynamics. In [33], the Minimum Pontryagin’s Principle is used to compute the UAV maneuvers. The minimum principle showed that the time-optimal trajectories are bang-bang in the thrust command. However, in the rotational rate control, they are bang-singular. In [34], numerical optimal control is used in order to solve a wide range of problems related to UAV systems.

#### 2.3.1. Velocity and Acceleration Constraints

Trajectory generation must not only describe the desired trajectory accurately, but must also have smooth kinematics profiles for increasing the precision and the durability of the system, maintaining higher tracking accuracy while avoiding exciting natural modes of the mechanical structure or servo control system [35]. To do so, the trajectory must satisfy certain constraints such as the vehicle’s physical limits, safety regulations and sensor specifications. In this work, we adopt a method of decoupling these limits into constraints on the trajectory’s velocity and acceleration. For the way-points at the corners, the velocities should be slowed down to zero, but the vehicle should pass through the way-points between the corners at a constant velocity. However, the acceleration should always be brought down to zero at every way-point.

#### 2.3.2. LQR Optimization Trajectory Generation

The approach used in this work falls under the second class. An LQR algorithm is applied for generating the position, velocity, and acceleration reference trajectories. As mentioned in Section 1, we are inspired by the work of Sanyal in [18]. The main differences between our approach and Sanyal’s are:

1. Our approach uses the snap of the quadrotor as the input control instead of the crackle. We believe that going up to the snap rather that the crackle assures to obtain trajectories with less curvatures. Besides, this would guarantee both continuity and smoothness of the reference trajectories at the way-points.
2. In our approach, the output vector is not constructed only from the position and the acceleration at the way-points only. The vehicle velocity is added. The aim is to (1) constrain the velocity at the way-points and (2) respect flying in corridors.
3. Our approach assumes an online generation of the quadrotor attitude with the use of a pointing direction and the IMU vector measurements. Hence, the proposed Kalman method in [18] is avoided.

The system states *x(t)* are given by:

(2) $$x\left(t\right)={\left[b\left(t\right)\;\dot{b}\left(t\right)\;\ddot{b}\left(t\right)\;\dddot{b}\left(t\right)\right]}^{T}$$

where *b(t)* is the position of the quadrotor in the 3D environment at instant *t*. The output *y(t)* is constructed such that the desired output *y_i_* at the way-points is given by:

(3) $$y\left(t\right)={\left[b_{i}\;v_{i}\;a_{i}\right]}^{T}$$

where *b_i_*, *v_i_* and *a_i_* are the position, the velocity and the acceleration constraints of the vehicle at the way-points, respectively.

The cost function for the position trajectory that is to be minimized is expressed as

(4) $$J=\sum_{i=1}^{N}{\left(y\left(t_{i}\right)-y_{i}\right)}^{T}S\left(y\left(t_{i}\right)-y_{i}\right)+\int_{t_{0}}^{t_{N}}\frac{1}{2}\left(x^{T}Qx+u^{T}Ru\right)dt$$

Subjected to the constraint equations:

(5) $$\left\{\begin{array}{c} \dot{x}\left(t\right)=Ax+Bu\\ y(t)=Cx(t) \end{array}\right.$$

where $A=\left[\begin{array}{cc} 0_{9\times 3} & I_{9\times 9}\\ 0_{3\times 3} & 0_{3\times 9} \end{array}\right],B=\left[\begin{array}{c} 0_{9\times 3}\\ I_{3\times 3} \end{array}\right],C=\left[\begin{array}{cccc} I_{3\times 3} & 0_{3\times 3} & 0_{3\times 3} & 0_{3\times 3}\\ 0_{3\times 3} & I_{3\times 3} & 0_{3\times 3} & 0_{3\times 3}\\ 0_{3\times 3} & 0_{3\times 3} & I_{3\times 3} & 0_{3\times 3} \end{array}\right].$ $I_{n\times n}$ is the identity matrix of dimension *n*, $t_{0}$ is the starting time, $t_{N}$ is the final time and the control input *u* is the snap. Let the Hamiltonian *H* be expressed as

(6) $$H=\frac{1}{2}x^{T}Qx+\frac{1}{2}u^{T}Ru+\lambda^{T}\left(Ax+Bu\right)$$

The conditions of optimality expressed in *H* are given below:

(7) $$\left\{\begin{array}{c} \dot{x}\left(t\right)=\frac{\partial H}{\partial \lambda }=Ax+Bu\\ \dot{\lambda }\left(t\right)=-\frac{\partial H}{\partial x}=-Qx-A^{T}\lambda \\ 0=\frac{\partial H}{\partial u}=Ru+B^{T}\lambda \\ 0=\frac{1}{2}\frac{\partial {\left(y\left(t_{i}\right)-y_{i}\right)}^{T}S\left(y\left(t_{i}\right)-y_{i}\right)}{\partial x(t_{i})}+\lambda \left(t_{i}^{+}\right)-\lambda \left(t_{i}^{-}\right) \end{array}\right.$$

where $t_{i}^{+}$ is the time instant $t_{i}$ when approached from times $t>t_{i}$ and $t_{i}^{-}$ s the time instant $t_{i}$ when approached from times $t<t_{i}$. Putting λ(t)=P(t)x(t)+η(t) then the governing equations for minimizing the cost *J* are:

(8) $$\dot{P}=-PA-A^{T}P-Q+PBR^{-1}B^{T}P$$

(9) $$\dot{\eta }=\left(-A^{T}+PBR^{-1}B^{T}P\right)\eta$$

(10) $$\dot{x}=\left(A^{T}+BR^{-1}B^{T}P\right)x-BR^{-1}B^{T}\eta$$

(11) $$u=-R^{-1}B^{T}\left(Px+\eta \right)$$

While the boundary condition ∀i∈W/{N} is

(12) $$P\left(t_{i}^{-}\right)=P\left(t_{i}^{+}\right)+C^{T}SC$$

(13) $$\eta \left(t_{i}^{-}\right)=P\left(t_{i}^{+}\right)-C^{T}Sy_{i}$$

At *i* = *N*, $P\left(t_{N}\right)=C^{T}SC$ and $\eta \left(t_{N}\right)=-C^{T}Sy_{i}$. The Equations (8) and (9) are solved backward in time starting at $t=t_{N}$ and updating the boundary conditions at every instant $t=t_{i},i<N$ as in Equations (12) and (13). The solutions obtained can be replaced in Equations (10) and (11) to get the vector state x(t) and u(t) at every instant *t*∈ [$t_{0},t_{N}$], respectively.

#### 2.3.3. Online Trajectory Re-planning

To cope with unmodeled obstacles, a lower trajectory re-planner module is required. The aim of this module is to run in several milliseconds to ensure the safety of quadrotor and keep it close to the global path while simultaneously avoiding unpredicted obstacles. The existing methods for quadrotor online trajectory re-planning can be mostly categorized as polynomials methods, sampling-based methods, and optimization methods [36]. Polynomials and splines are mostly suitable for re-planning since they are computationally efficient. However, they cannot provide time optimal policies, since polynomials are smooth by their own nature. Sampling-based methods such as RRT (Rapidly-exploring Random Tree) construct road maps from sampling free space and searching a graph map space. This can be achieved by computing edge cost and heuristic functions [37]. Such methods are inherently non-smooth and often do not satisfy the quadrotor’s dynamics constraints. Trajectory optimization methods take use of two types of cost functions: a smoothing function and a collision avoidance function. The sum of the two cost functions can be minimized then using optimizations approaches such as gradient-descent, Gauss-Newton by computing the gradient or the Jacobian of each cost function [38]. This method can be useful when dealing with cluttered environments. However, it suffers from the local minimum problem.

In our case, replanning is performed using an online Improved APF method [39]. This is widely used because its model is simple and elegant, and it is applicable for real-time implementation. Firstly, an attractive potential field is constructed at the target using the following expression:

(14) $$U_{att}\left(P\right)=\frac{1}{2}k_{att}.d^{2}\left(P,P_{g}\right)$$

where $d(P,P_{g})$ = $P_{g}-P$ is the Euclidean distance between the quadrotor’s position and the target position. $k_{att}$ is the attractive potential field constant.

The attractive force of the quadrotor in the attractive potential field is the negative gradient of $U_{att}$:

(15) $$F_{att}\left(P\right)=-\nabla U_{att}\left(P\right)=k_{att}.d\left(P,P_{g}\right)$$

Secondly, an improved repulsive potential field is constructed around the obstacles and can be defined as:

(16) $$U_{rep}\left(P\right)=\left\{\begin{array}{cc} \frac{1}{2}{\left(\frac{1}{d(P,P_{o})}-\frac{1}{d_{o}}\right)}^{2}d^{n}\left(P,P_{g}\right) & d\left(P,P_{o}\right)\leq d_{o}\\ 0 & d\left(P,P_{o}\right)>d_{o} \end{array}\right.$$

where $P_{o}$ is the position of the obstacle, $d_{o}$ is influence range of the repulsive potential field and *n* is an arbitrary real number which is greater than zero (for our case n=2).

The repulsive potential field is called improved since the distance correction factor $d^{n}\left(P,P_{g}\right)$ is added to the conventional repulsive potential field known in literature. This creates a balance between the two kind of forces especially in case where a rapid increase in the repulsive force occurs. In this manner, the repulsive force can be decreased gradually when the quadrotor is adjacent to the target. Most important of all, this force ensures that the overall potential field at the target is the global minimum. Thus, the improved repulsive force function is obtained as follows

(17) $$F_{rep}\left(P\right)=-\nabla U_{rep}\left(P\right)\left\{\begin{array}{c} F_{rep1}\left(P\right)+F_{rep2}\left(P\right)\;d\left(P,P_{o}\right)\leq d_{o}\\ 0\;d\left(P,P_{o}\right)>d_{o} \end{array}\right.$$

where $F_{rep1}$ and $F_{rrp2}$ are expressed as

(18) $$F_{rep1}\left(P\right)=k_{rep}\left(\frac{1}{d(P,P_{o})}-\frac{1}{d_{o}}\right)\frac{d^{n}\left(P,P_{g}\right)}{d^{2}\left(P,P_{o}\right)}$$

(19) $$F_{rep2}\left(P\right)=\frac{n}{2}k_{rep}{\left(\frac{1}{d(P,P_{o})}-\frac{1}{d_{o}}\right)}^{2}d^{n-1}\left(P,P_{g}\right)$$

**Figure 4:**
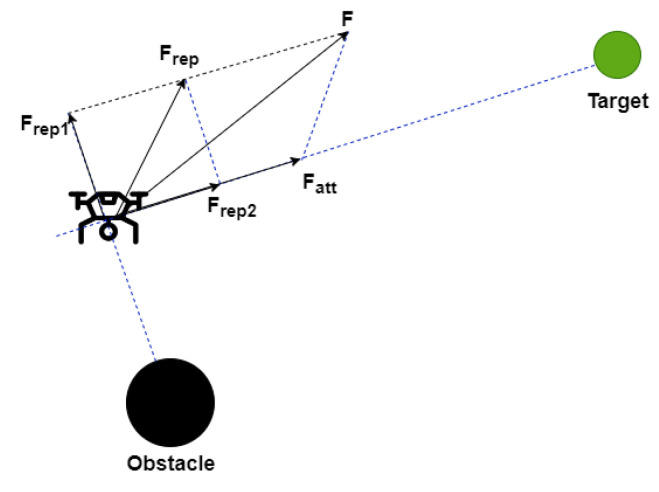
The improved resultant for model applied on the quadrotor.

The quadrotor moves toward the target in the joint action of the resultant force model (See Figure 4).

**Figure 5:**
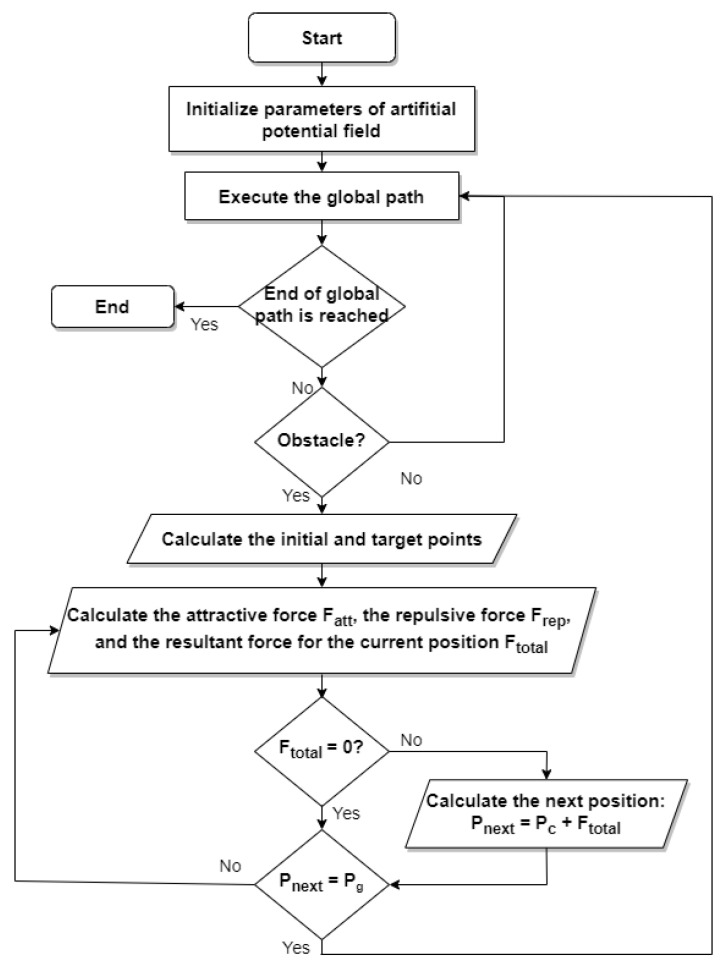
The flowchart of the proposed trajectory re-planning method based on the improved APF.

Figure 5 depicts a flowchart that explains the whole re-planning process. $P_{c}$ refers to the current position of the quadrotor.

### 2.4. Control and Trajectory Tracking

Once the optimal trajectory is generated as previously described, it is necessary to design a control strategy to track the reference trajectory. Many research studies have been conducted to construct such strategies for UAVs. Linear control systems such as Proportional-Derivative controller or LQRs are widely used to improve the equilibrium stability properties [40,41]. Nonlinear trajectory tracking techniques like backstepping [42] and sliding mode [43] are developed. However, most of these strategies exhibit singularities when dealing with complex rotational maneuvers. To avoid these singularities, geometric controllers are applied on dynamic systems evolving on nonlinear manifolds that are not globally identified with Euclidean spaces [19,44]. In this work, a geometric controller strategy, that uses the quadrotor dynamics expressed globally on the Special Euclidean SE(3) configuration manifold, is constructed to track predefined trajectories.

#### 2.4.1. Quadrotor Dynamics Model

**Figure 6:**
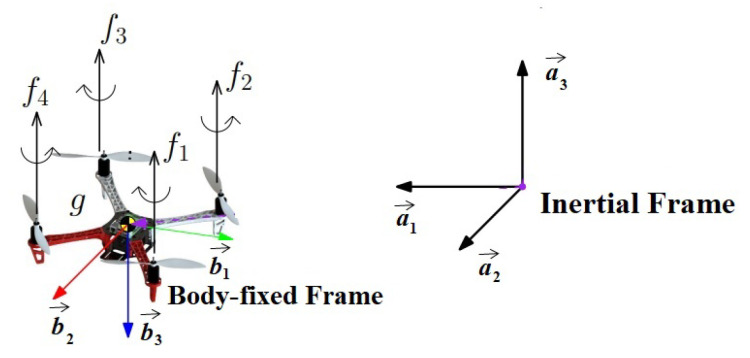
Quadrotor inertial and body-fixed frames.

A proper quadrotor mathematical model must be defined for designing a control system. In order to derive such a model, a set of coordinates systems for specifying the position, velocity, forces, and moments acting on the vehicle must be defined. Let the inertial frame be the surface of the earth and the body frame be fixed on the quadrotor rigid body as shown in Figure 6. $\left\{\vec{b_{1}},\vec{b_{2}},\vec{b_{3}}\right\}$ are the unit vectors along the body-fixed frame axis. $\left\{\vec{a_{1}},\vec{a_{2}},\vec{a_{3}}\right\}$ are the unit vectors along the inertial frame axis.

Using the Newton-Euler equations, the equation of motion of a quadrotor is derived. The model obtained is sufficiently reliable for simulating and controlling the vehicle behavior. It is given by

(20) $$\left\{\begin{array}{c} \dot{b}=v\\ m\dot{v}=fRe_{3}-mge_{3}\\ \dot{R}=R{\left[\Omega \right]}_{\times }\\ J\dot{\Omega }=-\Omega \times J\Omega +\tau \end{array}\right.$$

where $b\in R^{3}$ denote the position, $v\in R^{3}$ is the translational velocity in the inertial frame, *m* is the mass, *f* is the thrust magnitude, $e_{3}={\left[0,0,1\right]}^{T}$, *g* is the gravity, *J* is the moment of inertia, Ω is the angular velocity expressed in the body-fixed frame and τ is the external torque applied on the vehicle.

#### 2.4.2. Geometric Control Using Measurement Vectors

**Figure 7:**
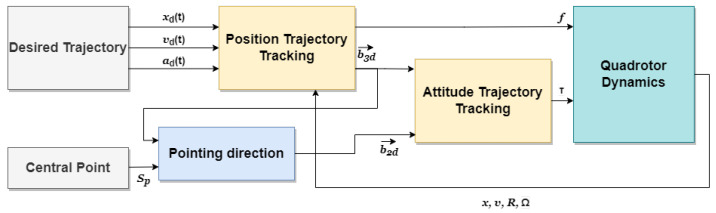
The structure of the geometric controller.

As stated in Section 1, we are inspired by the work in [19] to design a geometric controller for tracking the generated trajectory. However instead of using a heading direction and a thrust direction, inertial measurement vectors that are provided by a sensor are used to construct the vehicle attitude. In this way, new definitions of the errors between the real vectors and the desired ones are used (See Equations (28)–(30)). These errors are injected directly in the control law. Besides, the notion of a pointing direction is introduced instead of the heading direction. The controller structure is shown in Figure 7.

The quadrotor translational dynamics is controlled using the total thrust $fRe_{3}$. The magnitude of the total thrust *f* is directly controlled and its direction $Re_{3}$ is along the third body-fixed axis $b_{3}$. Hence, to obtain stabilized translational motion along a desired trajectory, the total thrust *f* and a desired direction of $b_{3d}$ are selected. A direction is required to complete the degrees of freedom of the desired attitude $R_{d}\in SO\left(3\right)$. Thus, a pointing direction vector $s^{c}$, which has to be corrected online using the desired direction $b_{3d}$, is chosen. The desired attitude is then obtained as $R_{d}=\left[b_{2d}\times b_{3d},b_{2d},b_{3d}\right]$ where $b_{2d}=s^{c}$. This desired attitude is followed by the control moment τ.

Section 2.3.2 results in the optimal desired position trajectories $x_{d}\left(t\right)$. The pointing directions at every instant $t\in [t_{0},t_{f}]$ are

(21) $$s\left(t\right)=\frac{S_{p}-x_{d}\left(t\right)}{\left|\right|S_{p}-x_{d}\left(t\right)\left|\right|}\;\forall t\in \left[t_{0},t_{f}\right]$$

The pointing directions can be corrected using the vector $b_{3d}\left(t\right)$ as shown in the expression below

(22) $$s^{c}\left(t\right)=\frac{s\left(t\right)-\left(s{\left(t\right)}^{T}b_{3d}\left(t\right)\right)b_{3d}\left(t\right)}{||s\left(t\right)-\left(s{\left(t\right)}^{T}b_{3d}\left(t\right)\right)b_{3d}\left(t\right)\left|\right|}$$

where $s^{c}\left(t\right)=b_{2d}\left(t\right)$, and $s^{c}\left(t\right)⊥b_{3d}\left(t\right)$. The vector $b_{3d}\left(t\right)$ is expressed as

(23) $$b_{3d}\left(t\right)=\frac{-k_{x}e_{x}-k_{v}e_{v}+m\left(ge_{3}+a\left(t\right)\right)}{\left|\right|-k_{x}e_{x}-k_{v}e_{v}+m\left(ge_{3}+a\left(t\right)\right)\left|\right|}$$

where $k_{x}$, $k_{v}$ are some positive constants, $e_{x}$ and $e_{v}$ are the tracking errors for the position x(t) and the velocity v(t), respectively. They are expressed as

(24) $$e_{x}=x-x_{d}$$

(25) $$e_{v}=v-v_{d}$$

The vector $b_{1d}\left(t\right)$ can be produced as

(26) $$b_{1d}\left(t\right)=b_{2d}\left(t\right)\times b_{3d}\left(t\right)$$

We assume that the quadrotor’s attitude is unknown for measurements (unavailable for feedback). We assume also that it is equipped with sensors that provide unfiltered vector measurements (in the body-fixed frame). Hence, the only variables available are the vector measurements which are denoted by $b_{i}$. We propose the following control law

(27) $$\tau =\Omega \times J\Omega +J{\dot{\Omega }}_{d}+Jz_{\rho }-Je_{\Omega 1}-Je_{\Omega 2}$$

where $z_{\rho }$, $e_{\Omega 1}$ and $e_{\Omega 2}$ are the errors and given by

(28) $$z_{\rho }=\sum_{i=1}^{k}\rho_{i}S\left(b_{id}\right)b_{i}$$

where $\rho_{i}>0$ and *k* is the number of measured vectors in the body frame (k=2 in our case). S(.) is the hat mapping transforms an vector to a skew-symmetric matrix: $S\left(.\right):R^{3}\to \mathrm{so}\left(3\right)$.

(29) $$e_{\Omega 1}=\Omega -\Omega_{d}$$

(30) $$e_{\Omega 2}=\Omega_{d}\times \Omega$$

The desired angular velocity is given by

(31) $$\Omega_{d}=Vex\left({\left[b_{1d},b_{2d},b_{3d}\right]}^{T}\left[{\dot{b}}_{1d},{\dot{b}}_{2d},{\dot{b}}_{3d}\right]\right)$$

with $Vex\left(.\right):\mathrm{so}\left(3\right)\to R^{3}$.

The proposed control law τ guarantees Almost Global Asymptotic Stability (AGAS) of the body attitude and angular velocity to their desired values. The proof is shown in the subsection below.

#### 2.4.3. Stability Analysis of the Proposed Control Law

The lemma in [45] is the key to the proof of almost asymptotic stability of the control law. Using the lemma, we can write

(32) $$z_{\rho }\equiv \sum_{i=1}^{k}\rho_{i}S\left(b_{id}\right)b_{i}=-2R_{d}^{T}\left(q_{0}^{e}I-S\left(q^{e}\right)\right)W_{\rho }q^{e},$$

where $W_{\rho }=-\sum_{i=1}^{k}\rho_{i}S{\left(r_{i}\right)}^{2}$ is a symmetric positive definite matrix according to the lemma. The tracking error $R^{e}=RR_{d}^{T}$ of the attitude corresponds to the unit quaternion errors

(33) $$Q^{e}=Q⊙Q_{d}^{-1}\equiv \left(q_{0}^{e},q^{e}\right)$$

where $Q\in S^{3}=\left\{Q=\left(q_{0},q\right)\in R\times R^{3}|q_{0}^{2}+q^{T}q=1\right\}$. Given the attitude dynamics in (20) and the control law (27), if we consider $\bar{\omega }=R_{d}e_{\Omega 1}$, where $e_{\Omega 1}$ is given in Equation (29), the closed loop dynamics are obtained as

(34) $${\dot{Q}}^{e}=\left[\begin{array}{c} {\dot{q}}_{0}^{e}\\ {\dot{q}}^{e} \end{array}\right]=\left[\begin{array}{c} -\frac{1}{2}{\left(q^{e}\right)}^{T}\bar{\omega }\\ \frac{1}{2}\left(q_{0}^{e}I+S\left(q^{e}\right)\right)\bar{\omega } \end{array}\right]$$

(35) $$\bar{\omega }=-\alpha \bar{\omega }-2\left(q_{0}^{e}I-S\left(q^{e}\right)\right)W_{\rho }q^{e}$$

Note that these dynamics are autonomous. Defining $x=(Q^{e},\bar{\omega })$ in the state space $X:=S^{3}\times R^{3}$, the above dynamics can be written in the form

(36) $$\dot{x}=f\left(x\right)$$

We can also note that (35) can be written as

(37) $$\dot{\bar{\omega }}=-\alpha \bar{\omega }+R_{d}z_{\rho }$$

**Theorem** **1.** *Consider the rigid body dynamics (20) with the control law (27) resulting in the closed loop attitude dynamics given by (34) and (35). Then, under assumptions of the lemma, the trajectories of (34) and (35) converges to the following subsets $S^{3}\times R^{3}$, given by $\Theta_{1}=\left(\pm 1,0,0\right)$ and $\Theta_{2}=\{\left(0,\pm v_{1},0\right),\left(0,\pm v_{2},0\right),\left(0,\pm v_{3},0\right)$}, where $v_{i}$ (i=1,2,3) are unit eigenvectors of $W_{\rho }$.*

- *The equilibrium set $\Theta_{1}$ is asymptotically stable with the domain of attraction containing $\Phi =\{X:=\left(Q^{e},\tilde{\omega }\right)\in S^{3}\times R^{3}|X^{T}PX<c\}$ with $P=diag(0,2W_{\rho },\frac{1}{2})$ and $c<2\lambda_{min}\left(W_{\rho }\right)$ and $\lambda_{min}\left(*\right)$ is the smallest eigenvalue of (∗)*
- *The equilibria defined by the set $\Theta_{2}$ are unstable and $\Theta_{1}$ is almost globally asymptotically stable.*

**Proof.**  Let us propose the Lyapunov function candidate

(38) $$V=2{\left(q^{e}\right)}^{T}W_{\rho }q^{e}+\frac{1}{2}{\bar{\omega }}^{T}\bar{\omega }$$

With $W_{\rho }$ symmetric positive definite. The time derivative of Equation (38) in view of (34) and (35) is expressed as

(39) $$\dot{V}=2{\left(q^{e}\right)}^{T}W_{\rho }\left(q_{0}^{e}I+S\left(q^{e}\right)\right)\bar{\omega }+{\bar{\omega }}^{T}\left(-\alpha \bar{\omega }-2\left(q_{0}^{e}I-S\left(q^{e}\right)\right)W_{\rho }q^{e}\right)$$

(40) $$\dot{V}=2{\bar{\omega }}^{T}\left(q_{0}^{e}I-S\left(q^{e}\right)\right)W_{\rho }q^{e}+{\bar{\omega }}^{T}\left(-\alpha \bar{\omega }-2\left(q_{0}^{e}I-S\left(q^{e}\right)\right)W_{\rho }q^{e}\right)$$

After simplification, we obtain

(41) $$\dot{V}=-\alpha {\bar{\omega }}^{T}\bar{\omega }$$

It can be noticed in (41) that $\dot{V}\leq 0$. In addition to the same equation, we have $\dot{V}=0$ only $\bar{\omega }=0$. Using the Equation (37) and $\bar{\omega }=0$, we get $z_{\rho }=0$. According to the lemma, this leads to the equilibrium sets ($q_{0}^{e}=\pm 1,q^{e}=0,\tilde{\omega }=0$) or ($q_{0}^{e}=0,q^{e}=\pm v_{i},\tilde{\omega }=0$) which corresponds to $\Theta_{1}$ and $\Theta_{2}$, respectively. It remains to show that $\Theta_{2}$ is unstable to complete the proof.

Let us define $\delta \equiv {\left(q^{e}\right)}^{T}\bar{\omega }$, and consider the dynamics of $q_{0}^{e}$ and δ around ($q_{0}^{e}=0,\bar{\omega }=0$) which corresponds to ($q_{0}^{e}=0,\delta =0$)

(42) $${\dot{q}}_{0}^{e}=-\frac{1}{2}\delta$$

(43) $$\dot{\delta }=-\alpha \delta -2\eta q_{0}^{e}$$

where η is an eigenvalue of $W_{\rho }$. Hence, the system expressed by (42) and (43) can be written as follows

(44) $$\dot{X}=\left[\begin{array}{cc} 0 & -\frac{1}{2}\\ -\alpha & -2\eta \end{array}\right]X=AX$$

With $X={\left[\begin{array}{cc} q_{0}^{e} & \delta \end{array}\right]}^{T}$ and $A=\left[\begin{array}{cc} 0 & -\frac{1}{2}\\ -\alpha & -2\eta \end{array}\right]$. The two eigenvalues of *A* are real and of opposite sign. The characteristic equation of *A* is given by $P\left(\lambda \right)=\lambda^{2}+2\eta \lambda -\frac{\alpha }{2}$. Hence, the roots are $\lambda_{1,2}=-\eta \pm \sqrt{\eta^{2}+\frac{\alpha }{2}}$. Since the equilibrium $\left(q_{0}^{e},\delta \right)=\left(0,0\right)$ is unstable, it can be concluded that $\Theta_{2}$ is unstable.

Given the proofs above and according to the Krasowsky-La Salle theorem, the equilibrium is almost globally asymptotically stable in this case. Hence, the proposed control law (27) guarantees AGAS. □

For the translational dynamics stability analyis, we can refer to [19].

## 3. Results

In this section, we present simulation results to validate the effectiveness of the proposed method. All simulation experiments were performed by Matlab on an HP laptop with a 2.90 GHz Intel® i7-10700 CPU and 32 Gb of RAM. The simulation have been carried out using a quadrotor with a mass of m=4.34 kg, and a moment of inertia of J=diag(0.820,0.0845,0.1377) kg m${}^{2}$. The quadrotor started at $t_{0}=0$ s at rest from a given position $b_{0}$ i.e, $x\left(t_{0}\right)={\left[b_{0},0_{3\times 1},0_{3\times 1},0_{3\times 1}\right]}^{T}$ with an initial attitude of simply $I_{3}$. Therefore, the vehicle’s initial pose was given by

(45) $$g_{0}=\left[\begin{array}{cc} I_{3} & b_{0}\\ 0 & 1 \end{array}\right]$$

### 3.1. In-Field Environment Setup

**Figure 8:**
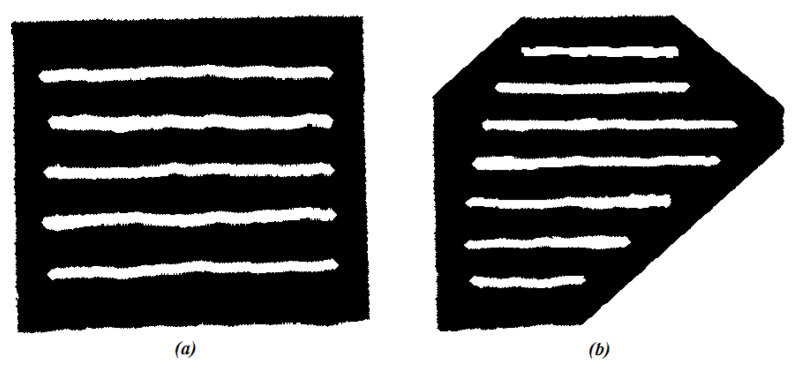
The vineyards scenarios in 2D: (**a**) flat rectangle, (**b**). tilted hexagon.

Vineyards terrains were selected to be our experimental environments. As stated before, those terrains are known by their steep slopes and limited room for maneuvering. We wanted the quadrotor to fly at relatively constant altitude between the vines and perform remote sensing. Since aerial imagery data was not available to build both real-life OGMs and DEMs, inflated artificial (generated) maps were used. The maps were hand-drawn using black and white colors, then converted to binary occupancy maps using Matlab. Two vineyard terrain scenarios were considered (See Figure 8). The first is a flat rectangle. The second is a tilted irregular hexagon.

### 3.2. Coverage Path Finding

**Figure 9:**
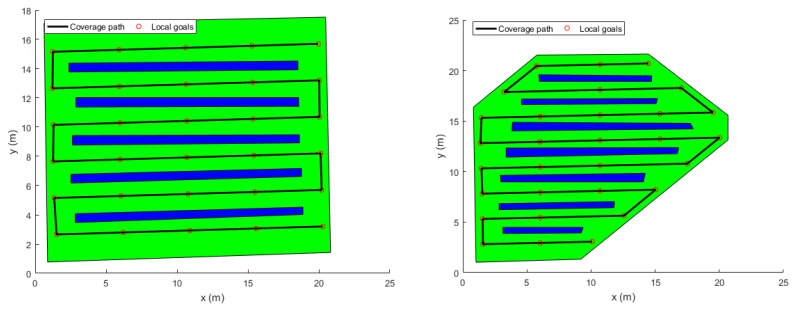
The generated coverage paths and way-points.

The binary occupancy maps above were fed to Matlab where the ISOA coverage path planning algorithm was run. As a result, a back and forth boustrophedon path was generated and way-points were extracted as explained in Section 2.2, as shown in Figure 9.

### 3.3. Trajectory Generation

**Figure 10:**
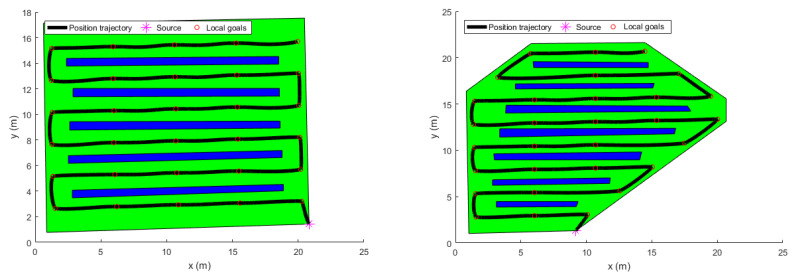
The generated optimal trajectories (2D View).

**Figure 11:**
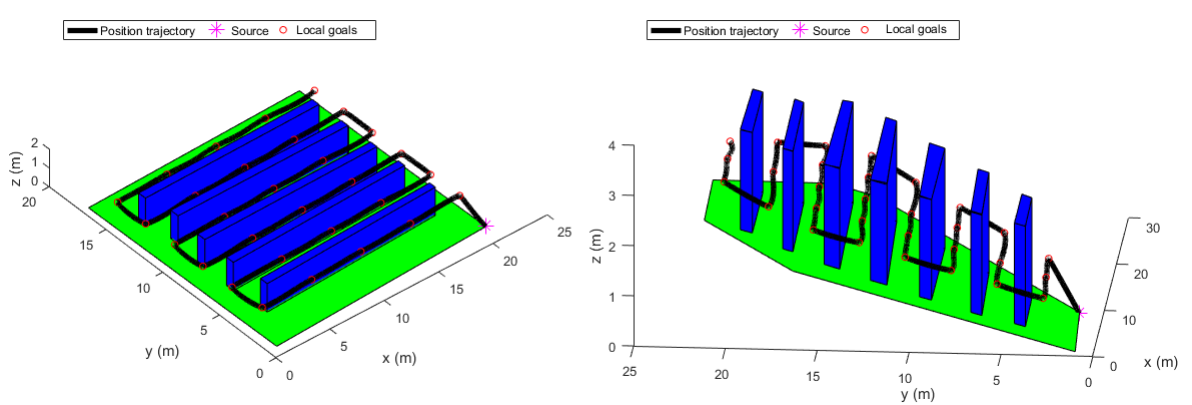
The generated optimal trajectories (3D View).

The extracted way-points were not fed directly to the trajectory generator since they contained geometric information only. Velocity and acceleration constraints were added to the way-points to keep the quadrotor flying in a corridor between the vines. To do so, a velocity of v=1 m/s was chosen. Besides, the time between the way-points was set to be 4 s. This information was used by the LQR trajectory generator. The resulting position trajectories of both fields, maneuvering around the way-points, are shown in Figure 10 and Figure 11.

### 3.4. Trajectory Tracking

In order to test the proposed trajectory tracking strategy, the flight position, velocity and acceleration information generated from the previous step were fed to the trajectory tracker. The pointing direction was chosen to be as expressed (21). Without loss of generality, $S_{p}$ was taken as the polygon centroid.

**Figure 12:**
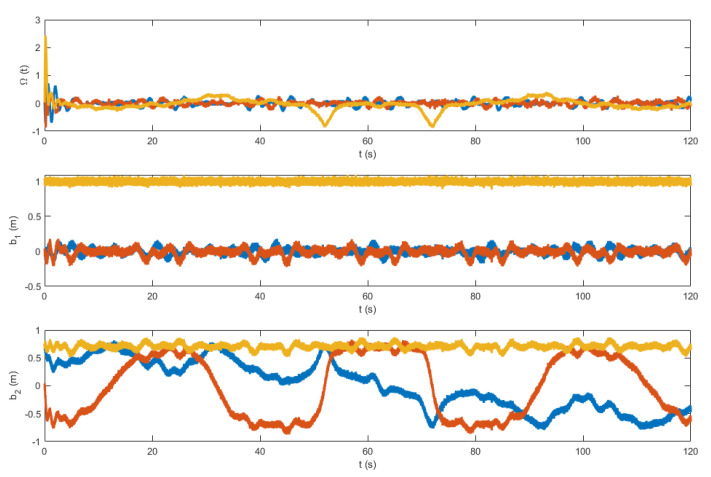
The vector measurements of the flight in terrain 1.

**Figure 13:**
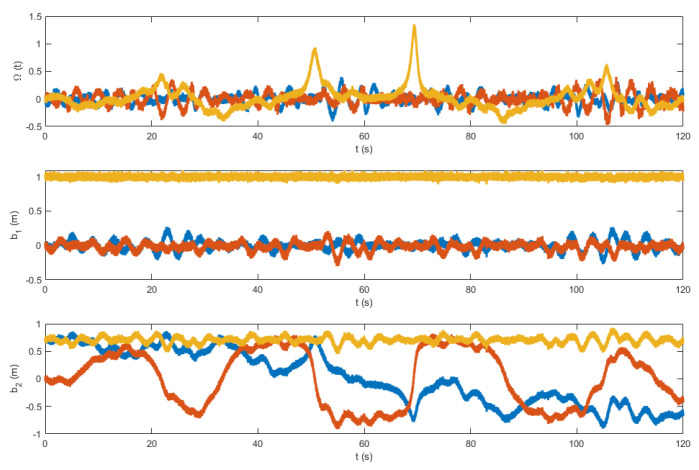
The vector measurements of the flight in terrain 2.

**Figure 14:**
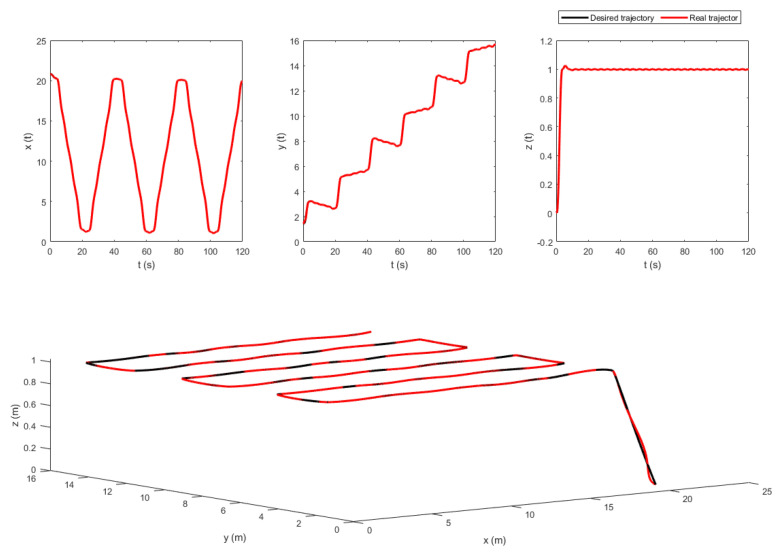
The tracking results of the flight in terrain 1.

**Figure 15:**
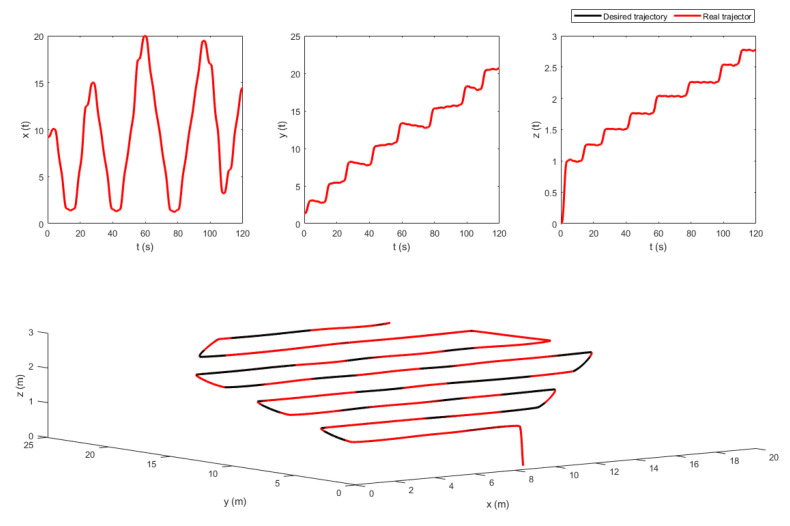
The tracking results of the flight in terrain 2.

**Figure 16:**
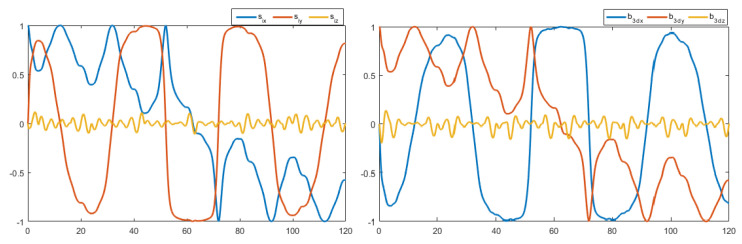
The pointing and thrust directions of the flight in terrain 1.

**Figure 17:**
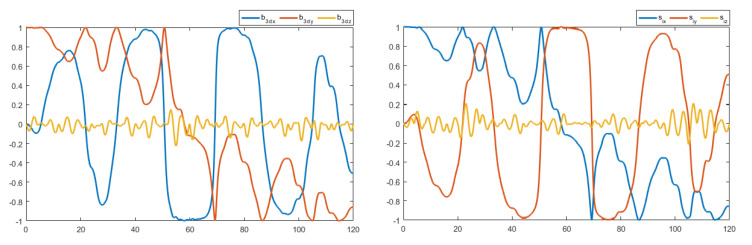
The pointing and thrust directions of the flight in terrain 2.

**Figure 18:**
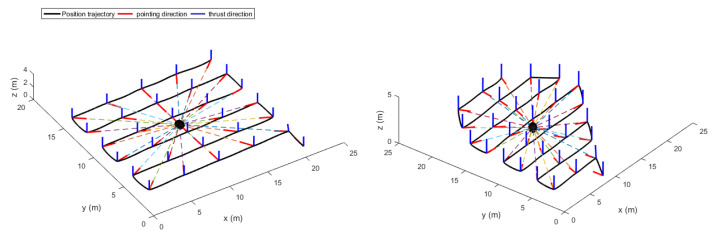
The pointing and the thrust direction vectors.

The control law in (27) contains the measurement vectors $b_{i}$ and Ω. These vectors were considered to be noisy. A zero mean white noise with variance of $5\times 10^{-4}$ was added. The resulting noisy measurement vectors are shown in Figure 12 and Figure 13. Figure 14 and Figure 15 show the tracking results of the generated flight position trajectories. Figure 16, Figure 17 and Figure 18 depict both the pointing and the thrust directions during the whole flights.

### 3.5. Trajectory Replanning

**Figure 19:**
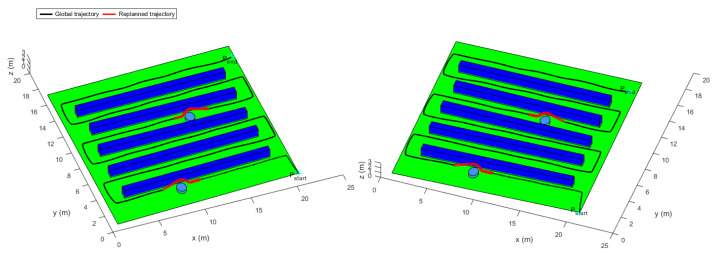
The global position and the replanned trajectories.

To evaluate the performance of the improved APF re-planning method, the first terrain was selected. Firstly, the quadrotor was assumed to be a particle. Secondly for sake of simplicity, we assumed no uncertainties in the simulated measured ranges between the vehicle and the obstacles. Besides, we supposed that the obstacles were in form of cylinders of equal sizes, located in the free environment (between the vines) and fixed at constant coordinates. The influence range of a single obstacle was fixed at $d_{det}=2.1$ m. Figure 19 shows the trajectory generated by the proposed method.

## 4. Discussion

In this paper, a complete solution for the GNC problem is proposed, which can provide stable and efficient navigation service for UAV autonomous flight mission in agricultural fields. As anticipated, the solution is based on a combination of existing algorithms. In path finding, Occupancy Grid Maps (OGMs) that maintain the environment information obtained from the terrain mapping process are adopted. Based on these maps, collision-free geometric coverage paths are generated according to the Iterative Structured Orientation algorithm (ISOA). In trajectory optimization, this paper proposes a Linear Quadratic Regulator (LQR) to generate minimum snap trajectories that satisfy position, velocity and acceleration constraints at the extracted local goals. In trajectory re-planning, an Improved Artificial Potential Field (APF) algorithm is used to enhance the safety of the trajectories when unknown obstacles are detected. In the trajectory tracking, a control law, which generates the attitude trajectories online using raw vector measurements, is implemented. The control law is used also to track the generated position and attitude trajectories while simultaneously pointing towards predefined direction along the whole flight mission.

In order to verify the effectiveness of the proposed method, a series of simulation experiments is conducted (See Section 3). As it can be seen in Figure 9, the ISOA generates efficiently complete optimal paths from a starting location to a final location. The back-and-forth generated path contains sharp turns which may contradict with the dynamics of the quadrotor. Hence, smoothing it represents a crucial step before sending any command to the vehicle. However, the main advantage of such algorithm is that it makes use of OGMs. These offer a powerful technique for representing the agricultural field environments and can be build from off-the-shelf sensors.

Figure 10 shows the position trajectory obtained from the LQR trajectory generator. It can clearly be seen that the continuous and smooth position trajectory maneuvers around the extracted waypoints successfully. This is achieved thanks to the LQR weighting diagonal terms *S*, *Q* and *R*. The choice of these terms is essential and plays a significant role in smoothing the trajectory features. However, choosing appropriate weighting terms is a challenging task.

**Figure 20:**
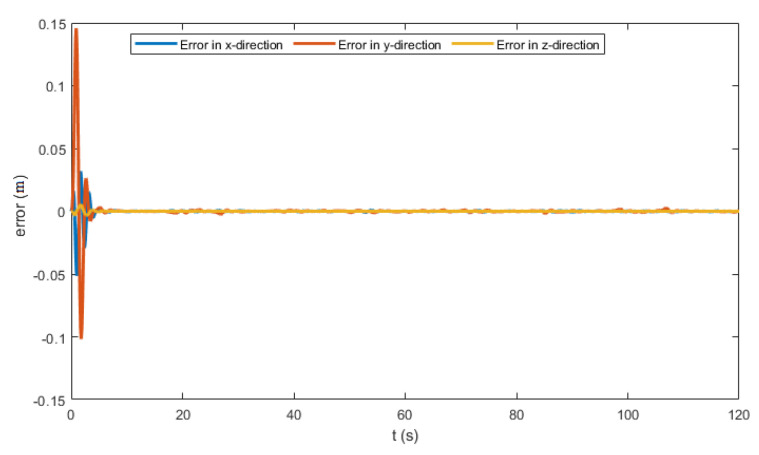
The position error profile for terrain 1.

**Figure 21:**
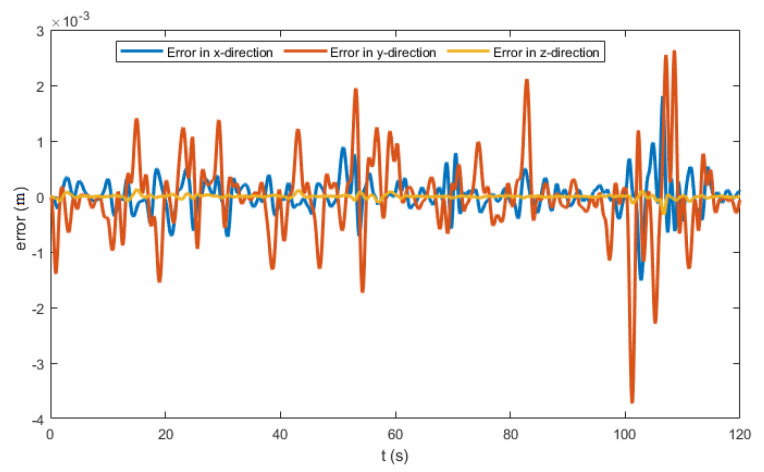
The position error profile for terrain 2.

The generated flight trajectories were tracked and analyzed. After flying 120 s, the quadrotor succeeded to track the desired trajectories despite the presence of white noise in the IMU. The comparison between the actual position/attitude trajectory (blue) and the desired position/attitude trajectory (red) is depicted in Figure 14 and Figure 15, showing that the two trajectories are identical, hence highlighting the effectiveness of the proposed control scheme to properly track the desired trajectories while fulfilling the pointing direction constraints not only at the local goals but at every position on the trajectory, as illustrated in Figure 18. In addition, a quantitative analysis of the two position trajectories along the x−, y− and z− axes is shown in Figure 20 and Figure 21 below. The absolute value of the maximum error, after stability is reached, can be calculated to be: $\sqrt{{\left(0.0010\right)}^{2}+{\left(-0.0026\right)}^{2}+{\left(-0.0001\right)}^{2}}=0.0028$ m for the first terrain and $\sqrt{{\left(0.0018\right)}^{2}+{\left(-0.0037\right)}^{2}+{\left(-0.0003\right)}^{2}}=0.0041$ m for the second one. As it can be noticed, the errors are in the order of $10^{-3}$ m. Such errors are suitable relative to the size of the terrains. Hence, this data validates the efficiency of the proposed tracking algorithm.

Figure 19 shows the selected vineyard terrain where static obstacles were added between the vine rows to obstruct the movement of the quadrotor in its global trajectory. The figure illustrates the quadrotor’s avoidance trajectory near the static obstacles. Note that the blue trajectory represents the desired re-planned trajectory obtained from the improved APF algorithm and the red trajectory represents the global trajectory obtained from the LQR trajectory generator. At every time step, the trajectory re-planner takes the position information of the global trajectory. Once the obstacle is sensed, it takes the position computed by the improved APF. It can be observed that the obstacle avoidance was performed vertically. This would prevent collision with the vine rows if the avoidance was done horizontally. In addition, it can be noticed that the relative distance maneuver remains greater than collision threshold that represents a physical collision. Above all, one can say that the quadrotor completes the avoidance mission safely.

However, although the combination of the algorithms has offered a complete and efficient solution for navigation in agricultural terrains, there are some issues that need to be improved. Since the selected algorithms are used in phases sequentially except the trajectory re-planning phase (See Figure 1), the output of a particular phase depends on the output of the previous one. Unreasonable outputs in a particular phase lead to intractable outputs in the next one. Consequently, the quality of the flight trajectories will be poor. In addition, the solution computational time depends on the dimensions of the mapped terrains. The more expansive the terrains are, the larger the computational time.

## 5. Conclusions

In this paper, an efficient guidance, navigation and control method for UAVs to complete 3D coverage flight missions in mapped agricultural fields is proposed. The method is mainly improved in four modules: coverage path searching, trajectory generation, trajectory re-planning, and trajectory tracking. In the coverage path searching module, a safe geometric coverage guiding path is generated on the artificial occupancy grid maps using the Iterative Structured Orientation method. The generated coverage path is optimized and smoothed using the trajectory generation module. The Linear Quadratic Regulation approach uses predefined velocity and acceleration constrained way-points from the former path to generate global minimum snap position trajectory with appropriate smoothness. In the trajectory re-planning module, the UAV modifies its global trajectory and the real-time local trajectory is generated to avoid the unpredicted obstacles. This can be achieved by the use of the Improved Artificial Potential Field method. The last module builds the geometric tracking controller on the Special Euclidean group. The aim of this module is tracking the predefined trajectory while simultaneously pointing toward the predetermined direction using the vector measurement provided by the vehicle inertial unit. Several numerical simulation results demonstrated the validity of the proposed GNC method. The presented approach offers an advantage to propose an innovative solution in the direction of autonomous navigation in agricultural fields since it is designed such that it complies with the peculiar features of the fields and the UAV. The approach also provides suitable performances in terms of optimal and efficient generation of trajectories and optimal tracking abilities for fully autonomous UAV. However, susceptibility to weather conditions like rain, fog and dust and maneuverability in winds and turbulence represent the main limitations of the approach. This latter becomes more challenging in large and unstructured agricultural environments where the vehicle localization and endurance issues are inherited.

Our future work will focus on extending the the proposed method to deal with external disturbances and to adapt to dynamic environments. Besides, although we carried out an extensive effectiveness evaluation of the method, the experiments were only carried out in simulation. Hence, the implementation and experimental validation of the method as presented herein is an important step towards this end.

## Figures and Tables

**Table 1:** The GNC problems solved by the mentioned references.

Reference	UGV	UAV	2D	3D	Coverage Path	Trajectory	Trajectory	Trajectory
No.					Planning	Optimization	Tracking	Re-Planning
[9]	X		X				X	
[11]	X		X		X			
[12]	X		X		X			
[13]	X		X		X	X		
[14]		X	X		X			
[15]		X	X		X	X		
[17]		X	X		X			
[16]		X		X		X	X	
